# The genome of *Leishmania panamensis*: insights into genomics of the *L.* (*Viannia*) subgenus.

**DOI:** 10.1038/srep08550

**Published:** 2015-02-24

**Authors:** Alejandro Llanes, Carlos Mario Restrepo, Gina Del Vecchio, Franklin José Anguizola, Ricardo Lleonart

**Affiliations:** 1 Centro de Biología Celular y Molecular de Enfermedades, Instituto de Investigaciones Científicas y Servicios de Alta Tecnología (INDICASAT AIP), Ciudad del Saber, Panamá, Panamá; 2 Facultad de Ciencias de la Salud Dr. William C. Gorgas, Universidad Latina de Panamá, Panamá, Panamá; 3 Department of Biotechnology, Acharya Nagarjuna University, Guntur, India

## Abstract

Kinetoplastid parasites of the *Leishmania* genus cause several forms of leishmaniasis. *Leishmania* species pathogenic to human are separated into two subgenera, *Leishmania* (*Leishmania*) and *L.* (*Viannia*). Species from the *Viannia* subgenus cause predominantly cutaneous leishmaniasis in Central and South America, occasionally leading to more severe clinical presentations. Although the genomes of several species of *Leishmania* have been sequenced to date, only one belongs to this rather different subgenus. Here we explore the unique features of the *Viannia* subgenus by sequencing and analyzing the genome of *L.* (*Viannia*) *panamensis*. Against a background of conservation in gene content and synteny, we found key differences at the genomic level that may explain the occurrence of molecular processes involving nucleic acid manipulation and differential modification of surface glycoconjugates. These differences may in part explain some phenotypic characteristics of the *Viannia* parasites, including their increased adaptive capacity and enhanced metastatic ability.

Leishmaniasis is a broad term describing several clinical presentations, ranging from mild cutaneous lesions to the life-threatening visceral form. The causative agents are kinetoplastid parasites of the genus *Leishmania*, transmitted to human by phlebotomine sandflies. During their life cycle the parasites exist in two life stages: a flagellated promastigote within the digestive tract of the sandfly vector and a non-motile amastigote infecting macrophages of the vertebrate host. The *Leishmania* (*Viannia*) subgenus encompasses several species distributed across the Neotropics, including *L. braziliensis*, *L. guyanensis*, *L. panamensis* and *L. peruviana*. This subgenus was defined by Lainson and Shaw^1^ based on differences in the site of propagation of promastigotes inside the digestive tract of the insect vector. These species primarily cause cutaneous leishmaniasis, but the parasites may occasionally migrate to nasopharyngeal tissues leading to highly disfiguring lesions in a presentation known as mucocutaneous leishmaniasis. This clinical form is exclusively associated with species of the *L.* (*Viannia*) subgenus. However, it is thought to be caused by a variety of factors that may contribute to enhance the metastatic ability of the parasites^2^, including their infection by a specific retrovirus called *Leishmania* RNA virus (LRV)^3^.

The genomes of Old World *Leishmania* species such as *L. major* have 36 chromosomes, whereas those of *L.* (*Viannia*) species have only 35, due to the fusion of chromosomes corresponding to 20 and 34 in *L. major*^4^. All *Leishmania* genomes sequenced to date have ~8,000 protein-coding genes which lack introns and are organized in relatively large polycistronic units called directional gene clusters (DGCs)^5^. Mature mRNA coding for each gene is formed by 5'-trans-splicing coupled to 3'-polyadenylation of the gene immediately upstream in the polycistronic primary transcript^6^. Gene expression is not primarily regulated at the level of transcription, but rather post-transcriptionally at the levels of mRNA stability and translation^7^.

*L. braziliensis* is the only species of the *Viannia* subgenus whose genome has been completed^8^. This study highlighted several differences at the genomic level as compared to other *Leishmania* species, such as the presence of potentially active mobile elements and several genes possibly involved in an RNA-mediated interference (RNAi) machinery. RNAi activity was later experimentally confirmed in *Viannia* parasites, and further proposed to serve as a protective mechanism against the effect of transposable elements and viruses such as LRV^9^. Here we present a high quality draft of the *L.* (*Viannia*) *panamensis* genome. This is the second genome sequenced for species of the *Viannia* subgenus, therefore allowing us to conduct a more detailed study of subgenus-specific features at the genomic level. *L. panamensis* is the main causative agent of tegumentary leishmaniasis in Panama^10^ and Colombia^11^, and is responsible for a relatively large number of cases in other neighboring countries. In Panama, there are about 3,000 new cases per year, 5% of which progress to the mucocutaneous presentation.

## Results

### Genome sequencing and assembly

The PSC-1 strain (MHOM/PA/94/PSC-1) of *L. panamensis* was selected to sequence its genome as it has been used as a reference strain for previous epidemiological studies in Panama^12^. We obtained two 454 read libraries with a total of 4 million reads and an estimated 30X coverage of the genome, one with single (shotgun) reads and the other with mate-paired reads and a median insert size of 8 kb. Additionally, a library of paired-end 100-bp Illumina reads was obtained, in this case not intended for *de novo* assembly, but for assembly validation, error correction and chromosome or gene copy number analyses. The 454 and Illumina read libraries were deposited in the Sequence Read Archive (SRA) under the accession codes SRX681913, SRX681914 and SRX681983.

*De novo* assembly of the 454 reads by using Newbler^13^ yielded 108 scaffolds with an N50 size of 674 kb, a total size of 30.9 Mb and 2.29% of N (~600 gaps) (Supplementary Table 1). The assembly was validated by using REAPR^14^, a program that uses the information contained in mate-paired reads to detect scaffolding errors and to break the original scaffolds at those points. The program flagged 81 errors, producing a fragmented assembly with a re-calculated N50 size of 555 kb. The REAPR-validated scaffolds were pooled together with the unassigned contigs and submitted to iCORN^15^ for correction of 454 pyrosequencing errors, resulting in several thousand corrections during 10 iterations (Supplementary Fig. 1).

A total of 140 fragments could be further oriented and contiguated into 35 pseudochromosomes by using ABACAS^16^ and the genome of *L. braziliensis* as a reference. Several relatively short fragments could not be assembled into pseudochromosomes due to conflicting or low sequence similarity. Twelve percent of these fragments matched kinetoplast DNA (kDNA) sequences and were not considered further. The remaining fragments have a concatenated size of 350 kb and are generally too short to span complete genes, but some of them are indeed fragments of genes organized in tandem arrays (further discussed ahead). It is important to stress that the scaffolds and not the individual contigs were submitted to ABACAS, so that the original assembly information surviving REAPR would be preserved. This served to detect several rearrangements between our assembled pseudochromosomes and the corresponding *L. braziliensis* chromosomes (Supplementary Figure 2). Comparisons regarding synteny and the chromosomal position of genetic elements throughout this article refer to regions assembled *de novo* for *L. panamensis*. The assembled pseudochromosomes were deposited in GenBank under the accession codes CP009370 to CP009404.

### Annotation of protein-coding and non-coding RNA genes

RATT^17^ was used to transfer the gene models annotated in the *L. major* and *L. braziliensis* genomes to the *L. panamensis* pseudochromosomes. Approximately 95% of the *L. braziliensis* annotated gene models could be transferred either completely or partially, compared to only 65% of those from the more distant *L. major* genome. However, 6% of the genes from *L. braziliensis* were transferred to regions apparently non-syntenic, although gene order is rather conserved within them. With a few exceptions, the synteny in our assembly matches that of the *L. major* genome. These regions account for many of the rearrangements we previously found during contiguation with ABACAS, together comprising nearly 490 annotated gene models (Supplementary Data 1). The relatively high sequence similarity between corresponding genes in these regions, as well as the conserved synteny between our assembly and the *L. major* genome, suggest that some of these segments may have been incorrectly placed in the *L. braziliensis* assembly.

Gene models transferred by RATT and those predicted *de novo* on the basis of codon usage bias were combined and manually curated, resulting in 7,748 predicted protein-coding genes and 185 suspected pseudogenes, distributed in 132 DGCs (Table 1 and Supplementary Fig. 3a). We were also able to annotate most of the non-coding RNA genes previously described in *L. major*, including the vast majority of tRNA genes^18^ and those encoding the 28S rRNA (LSU-α, β, γ, δ, ε, and ζ)^19^. Likewise, most of the clusters of small nucleolar RNA genes (snoRNA) detected in *L. major*^20^ are conserved in *L. panamensis*, with a few exceptions that appear to have degenerated to a point in which the typical signatures of these molecules are no longer recognizable. Interestingly, we found two genes encoding H/ACA-like snoRNAs in chromosome 35 that are similar to two *T. brucei* genes (TB10Cs5H2 and TB10Cs5H3). This cluster might be a *Viannia*-specific trait, as it is absent from *L. major* but present in *L. braziliensis* —although currently not annotated.

### Functional and comparative analysis of protein-coding genes

**Figure 1:**
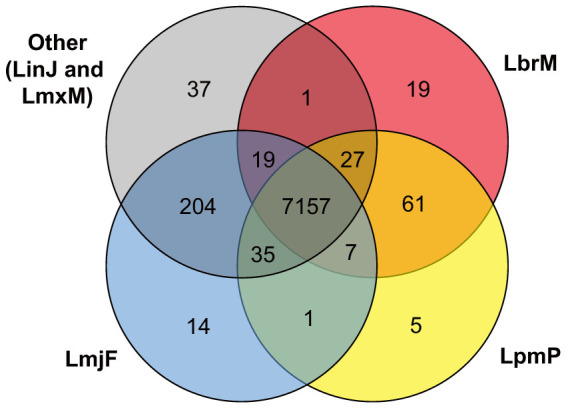
Distribution of ortholog groups among species of the *L.* (*Leishmania*) and *L.* (*Viannia*) subgenera. Simplified Venn diagram showing the number of conserved ortholog groups among *L. major* (LmjF), *L. infantum* (LinJ), *L. mexicana* (LmxM), *L. braziliensis* (LbrM) and *L. panamensis* (LpmP). To emphasize the differences between species of the *L.* (*Leishmania*) subgenus (left) and the *L.* (*Viannia*) subgenus (right), the ortholog groups from *L. infantum* and *L. mexicana* were merged into a single circle colored gray, regardless if they are shared between these two species or exclusively found in each of them. Also, this type of diagram excludes two relationships, namely the groups shared between *L. major* and *L. braziliensis*, and those shared between *L. panamensis* and the merged species. No groups were found in the latter category, whereas only one group is exclusively shared between *L. major* and *L. braziliensis*.

Functional analysis of the predicted gene models allowed us to ascribe a putative function to nearly 300 genes transferred from *L. braziliensis* and *L. major* without functional annotation. Running OrthoMCL on the annotated gene models resulted in 7,157 ortholog groups with orthologs in all *Leishmania* species included in OrthoMCL-DB (version 5), with only ~430 groups differing in at least one species (Fig. 1 and Supplementary Data 2). As expected, the number of groups shared between pairs of species of the same subgenus is larger than the number of groups shared between pairs from different subgenera.

Considering all *Leishmania* species analyzed, genes found to be differentially present or absent in *L. braziliensis* are similar to those in *L. panamensis*. One of the most discussed examples are the genes suspected to be involved in the RNAi machinery, including those encoding a Dicer-like endonuclease (*DCL1*) (LbrM.23.0390/LpmP.23.0400) and an Argonaute-like protein (*AGO1*) (LbrM.11.0360/LpmP.11.0590). Conversely, an example of genes previously found to be absent in *L. braziliensis* are those of the *HASP*/*SHERP* locus^8^. This locus encodes a family of surface proteins differentially expressed throughout the *L. major* life stages, which have been shown to be critical for parasite differentiation in the sandfly vector^21^. It was later demonstrated that *L. braziliensis* has a stage-regulated HASP ortholog (*oHASP*), divergent in sequence but with similar biochemical properties to that of *L. major*^22^. This *L. braziliensis* gene (LbrM.23.1120) also has a relatively similar ortholog in *L. panamensis* (LpmP.23.1160). The corresponding loci are syntenic to those from *L.* (*Leishmania*) species, but unlike the *L. major* locus, we found no evidence supporting the presence of several copies in *L.* (*Viannia*) (see Gene copy number variation analysis).

Divergence in orthologous genes might also explain other differences than have been found when conducting comparative studies among species of *L.* (*Leishmania*) and *L.* (*Viannia*), especially with respect to surface proteins. For example, the *PSA-2*/*GP46* gene encoding a major promastigote surface antigen could not be found in *L.* (*Viannia*) by using antibodies and hybridization probes specific for *L.* (*Leishmania*)^23^. The authors attempted to explain the putative loss of this gene in *L.* (*Viannia*) by chromosomal deletion, but also suggested the possibility of rapid evolution leading to sequence divergence. The latter seems to be the correct explanation, since *L. braziliensis* and *L. panamensis* share two orthologs (LbrM.12.0760 and LpmP.12.0760) with low sequence similarity to the *PSA-2* genes from *L.* (*Leishmania*) but located in approximately the same position in chromosome 12. In addition, the sequence similarity is higher in the region occupied by the leucine-rich domains (LRR) in the genes from *L.* (*Leishmania*), which have been demonstrated to be critical for the interaction of PSA-2 with the macrophages of the mammalian host^24^. Evidence supporting rapid differential evolution was in fact reported later for this family^25^, although LbrM.12.0760 was not considered in that study.

Another example of divergent orthologous genes related to surface components are those involved in the synthesis and modification of the lipophosphoglycan (LPG). LPG is a major surface glycoconjugate in *Leishmania*, and is considered to be a critical factor for parasite survival both in the insect vector and the mammalian host^26^. It is commonly modified by addition of carbohydrate side chains that vary significantly among life cycle stages and species. Genes encoding enzymes involved in LPG side chain modification therefore exhibit high variability among species, with a tandem array in chromosome 2 and several loci located in subtelomeric regions^27^. The largest family, *SCG*, encompasses several genes encoding β1,3-galactosyltransferases involved in the addition of galactose residues to the LPG side chains. In *L. major*, these modifications are thought to promote attachment of the non-infective promastigotes to the sandfly midgut, whereas further capping of the side chains with arabinose residues causes the highly infective forms to detach^28^. We were able to find several loci for *SCG* genes in *L. panamensis*, including a gene in the tandem array of chromosome 2 that is very similar to its ortholog in *L. braziliensis*, but very different to those from *L.* (*Leishmania*). However, we did not find the genes encoding the β1,2-arabinosyltransferases (*SCA1* and *SCA2*) involved in arabinose capping of the LPG side chains. This is consistent with the finding that LPG side chains in *L. braziliensis* are not apparently modified with arabinose, but with glucose^29^.

In agreement to previous studies^8^, pseudogene formation appears to be the main cause of gene loss in *L. panamensis*. Most genes annotated as pseudogenes appear to be deteriorated coding sequences, with intact orthologs in other *Leishmania* species. We noticed a relatively high number of pseudogenes in *L. panamensis* as compared to *L. major* or *L. infantum*. Although this finding may be attributed to errors introduced by next-generation sequencing, the *L. braziliensis* genome, which was completed by using the more accurate traditional Sanger sequencing, also appears to have a relatively large number of pseudogenes^30^. Pseudogenization seems to be frequent in duplicated genes, in which case a duplicated copy is converted into a pseudogene by diversification and eventual deterioration. A relevant example is an adenine phosphoribosyltransferase gene in *L. braziliensis* (LbrM.26.0120), previously suggested to have arisen by tandem duplication^8^, whose ortholog in *L. panamensis* appears to have become a pseudogene (LpmP.26.0130). We also noticed a few examples of gene loss by apparent deletion, such as a gene encoding a guanine nucleotide-binding protein present in all the species included in this study (LbrM.14.0740 in *L. braziliensis*) but absent from *L. panamensis* with no detectable deteriorated sequence.

**Figure 2:**
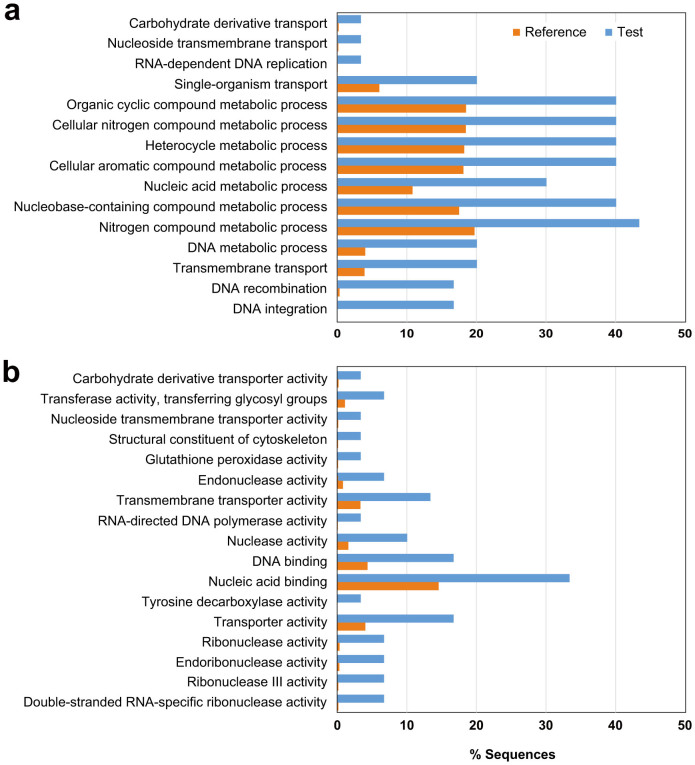
Gene Ontology (GO) terms overrepresented in the “*Viannia*-specific” genes. GO enrichment analysis for the *L. panamensis* genes with corresponding orthologs in *L. braziliensis* (test) against all the *L. panamensis* annotated genes (reference). GO terms found to be overrepresented with a *p*-value below 0.05 are shown ordered by *p*-values in ascending order for molecular processes (a) and cellular functions (b).

To better emphasize the differences between the two subgenera, a one-tailed Fisher's exact test for Gene Ontology (GO) term enrichment was performed for the *L. panamensis* genes uniquely shared with *L. braziliensis* —excluding those suspected to be separately clustered due to high sequence divergence— using the whole theoretical proteome of *L. panamensis* as the reference set (Fig. 2). Many of the enriched GO terms are associated with functions or processes involving nucleic acids, which in these species is due to the presence of an active RNAi pathway (endoribonuclease activity and double-stranded RNA-specific ribonuclease activity) and possibly active mobile elements (DNA integration, DNA recombination and RNA-dependent DNA replication).

Several terms related to transmembrane transport are related to a gene putatively encoding an equilibrative nucleoside transporter (LbrM.28.0580/LpmP.28.0570) with no direct ortholog in *L.* (*Leishmania*) species. This gene has only weak similarity to the four nucleoside/nucleobase transporters (NT1 to NT4) previously characterized in all *Leishmania*^31^ and also predicted to be present in *L. panamensis*. This plethora of genes coding for nucleoside/nucleobase transporters may be due to the fact that, like most parasitic protozoa, *Leishmania* is not able to synthesize purines *de novo* and therefore relies upon the salvage of purines from their hosts^32^. The occurrence of several processes involving manipulation of nucleic acids in *L.* (*Viannia*) could result in an increased demand of nucleosides, which may be in part fulfilled by acquisition via this additional transporter.

Another interesting finding is the enrichment of GO terms related to metabolic processes involving nitrogen compounds, in part associated with an additional gene coding for a glutathione peroxidase, as well as a gene putatively encoding a tyrosine/DOPA decarboxylase. Although *Leishmania* species have several enzymes with peroxidase activity, the presence of an additional glutathione peroxidase gene in *L.* (*Viannia*) (LpmP.26.0780/LbrM.26.0810) suggests an enhanced resistance to oxidative stress in these parasites, another factor that has been implicated in the development of metastatic clinical presentations^2^. Conversely, the tyrosine/DOPA decarboxylase gene (LbrM.30.2460/LpmP.30.2430) putatively codes for a function apparently exclusive to *L.* (*Viannia*), since the orthologous loci in *L.* (*Leishmania*) species are likely to be pseudogenes. Theoretically, such an enzyme would mediate the conversion of tyrosine into tyramine. This would be an alternative way of processing tyrosine, because *Leishmania* can only convert tyrosine into 4-hydroxyphenylpiruvate by using two different aminotransferases^33^. Additionally, this enzyme can theoretically mediate the conversion of dihydroxyphenylalanine (DOPA) into dopamine. Inferences on the biological relevance of such an enzyme in these parasites would require further experiments.

### Repetitive sequences and mobile elements

*De novo* detection of repetitive sequences resulted in over 220 repeat families, with repeat units of length ranging from 50 to 1,200 bp —excluding protein domains and other repetitive regions within coding sequences. Bases in repetitive sequences represent ~4% of the total base content of the *L. panamensis* genome, with many families uniformly distributed across chromosomes (Supplementary Fig. 3b). We identified 70% of all repetitive sequences as short interspersed degenerated retroelements (SIDERs), 31% from the SIDER1 subfamily and 41% from the SIDER2 subfamily. The abundance of these extinct retroposons in *Leishmania* genomes has been attributed to their role in regulation of gene expression^34^ and, more recently, to their ability to participate in recombinational events leading to genetic amplification^35^. We could also confirm the presence of telomere-associated mobile elements (TATEs) in the *L. panamensis* genome. Unlike SIDERs, TATEs belong to a family of putatively active mobile elements described for the first time in *L. braziliensis* genome^8^. It is important to mention that we found repeat families similar or related to TATEs located in internal positions of chromosomes, both in *L. panamensis* and *L. braziliensis*, thus indicating that these elements may not be associated specifically with telomeres as suggested by their name.

In addition, we found several relatively large repeat families with predicted protein-coding genes, later clustered in OrthoMCL groups that exclude *L.* (*Leishmania*) species. An example is OG5_141602, which contains hypothetical proteins from different chromosomes of *L. braziliensis*, *L. panamensis* and *Trypanosoma* spp. At least two of the proteins from the *L.* (*Viannia*) species in this group (LbrM.05.0960 and LpmP.19.1310) were predicted to have reverse transcriptase (RNA-dependent DNA polymerase) domains. These proteins have vague similarity to those from retroposons of the *ingi*/L1Tc clade of *T. brucei gambiense*, *T. congolense* and *T. cruzi*^36^. Although the repeats we found in *L.* (*Viannia*) species are shorter and lack other features previously described in such retroposons, we consider the presence of these hypothetical proteins to be a unique and intriguing trait of this subgenus. Furthermore, the region containing the gene LbrM.05.0960 (LpmP.05.0960 in *L. panamensis*) is flanked by two inverted copies of the *MST* gene, which encode a 3-mercaptopyruvate sulfurtransferase involved in the *Leishmania* defense against oxidative stress^37^. The length of the region is conserved in *L.* (*Leishmania*) species, but only one copy of the *MST* gene is present in these species.

### Variations in chromosome somy

**Figure 3:**
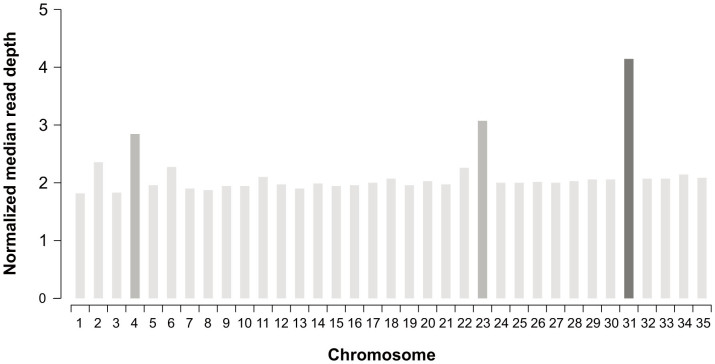
Estimates of chromosome somy for *L. panamensis* strain PSC-1. Normalized median read depth is plotted for each *L. panamensis* chromosome. Bars are shaded according to estimated chromosome somy: light gray (disomic), medium gray (trisomic) and dark gray (tetrasomic).

Alignments of the Illumina reads to the assembled pseudochromosomes were used for ploidy or chromosome somy estimations. Despite local spikes associated with repetitive features, distribution of read depth is relatively uniform along the sequence of all chromosomes (Supplementary Figure 4). Median read depth does not appear to be globally affected by local variations in depth of coverage or GC content bias (Supplementary Figure 5). Several studies in our laboratory have shown that the PSC-1 strain is mostly diploid, thus we arbitrarily assigned the most frequent value of median read depth within all chromosomes to a disomic state. This value was used to compute a normalized median read depth for each chromosome, which was considered to be an estimation of its somy (Fig. 3 and Supplementary Data 3). Most chromosomes seem to be disomic, with the exception of chromosomes 4 and 23, which appear to be trisomic, and chromosome 31, which seems to be tetrasomic. Chromosome 31 has been previously found to have an unusually larger somy in *Leishmania*; in fact, this is the only chromosome that has been found to be supernumerary in all *Leishmania* species in previous studies^30^,^38^. As reported by Rogers *et al*.^30^, we also noticed irregular values for median read depth in some of the smallest chromosomes, suggestive of chromosomes that are not fully disomic or trisomic. However, this situation is likely to be a consequence of mosaic aneuploidy^39^, a phenomenon recently described for *Leishmania*, characterized by a variation in the somy of the same chromosomes among cells within the population.

**Figure 4:**
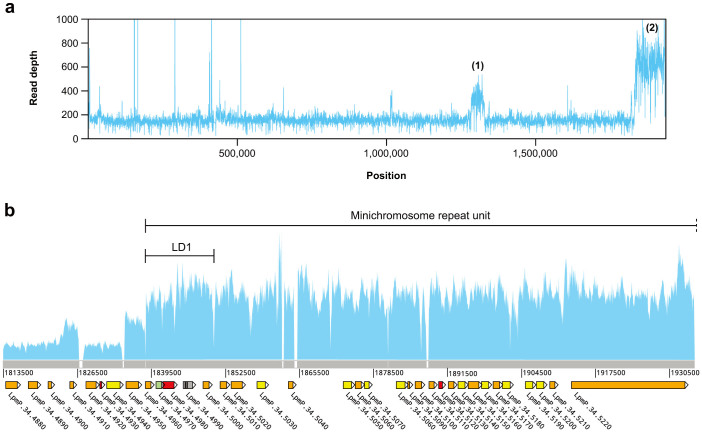
Relatively large local changes in read depth suspected to be caused by amplification in the *L. panamensis* genome. (a) Distribution of read depth along chromosome 34, showing two relatively large regions with a uniform increase in read depth, labelled (1) and (2), respectively. (b) Region (2) in detail. Protein-coding genes are represented along a segment of the chromosome sequence, with white boxes indicating assembly gaps. Genes are colored according the knowledge of their function (Supplementary Fig. 3a). Also indicated are the canonical LD1 region and the larger sequence suspected to form the two inverted repeats of the minichromosome (see text). The dashed line in one of the two ends of this sequence indicates that it extends to the telomeric region, which could not be properly assembled.

We also noticed two relatively large regions with a uniform increase in read depth in chromosome 34, spanning approximately 45 and 100 kb, respectively (Fig. 4a). These regions are likely to be amplified, either duplicated within the chromosomes or as extrachromosomal elements. The first one encompasses ten genes of unknown function, with the exception of one (LpmP.34.3370) putatively encoding a protein of the *SMC* (structural maintenance of chromosome) family. However, the second one contains the LD1 region, a well-studied region prone to numerous types of amplifications in *Leishmania*^40^. The “canonical” LD1 region was defined as a 27-kb segment from chromosome 35 of an *L. infantum* strain, which occurs as two inverted repeats in an amplified circular episomal element^41^. However, many different types of amplicons containing this region have been described^42^, all with an inverted repeat dimer organization. The amplification found in this work resembles a 245-kb linear minichromosome previously described in *L. braziliensis*^43^, consisting of two inverted repeats of ~120 kb arranged so that the original telomeric region of chromosome 34 is placed at both ends (Fig. 4b). The abundance of LD1 amplifications among *Leishmania* species has been attributed to the presence of a *BT1* gene encoding a biopterin transporter (LpmP.34.4980 in *L. panamensis*), which may contribute to an improved capture of pterins when it is amplified. This particular type of minichromosome may be considered a *Viannia*-specific feature, although it is not present in all strains or in all lineages of the same strains in *L. braziliensis*. It has been demonstrated that the presence of the minichromosome in *L. braziliensis* favors the survival of parasites and improves their infectivity in macrophages and in the sandfly vector^44^.

### Gene copy number variation analysis

**Figure 5:**
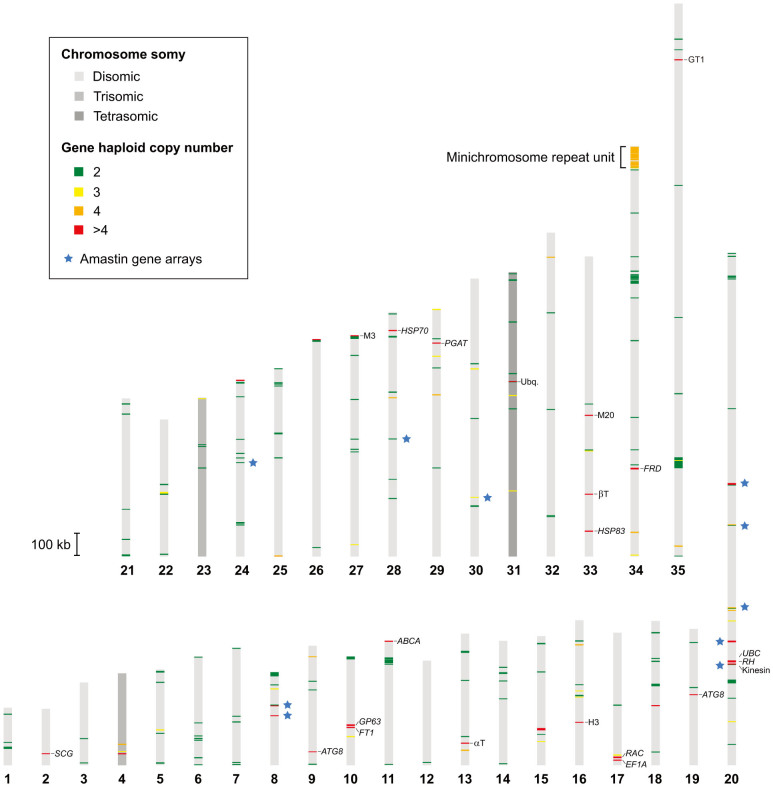
Overview of chromosome somy and gene copy number variation in *L. panamensis* strain PSC-1. Distribution of putative gene arrays along chromosomes, with color scales indicating the estimated chromosome somy and haploid copy number, respectively. Arrays with more than four estimated copies whose function could be ascribed are labeled. Abbreviations: αT, α-tubulin; βT, β-tubulin; H3, histone H3; M3, peptidase M3; M20, peptidase M20; Ubq., ubiquitin; the rest of the acronyms are gene names (Table 2).

The haploid copy number of protein-coding genes was estimated from the alignments of Illumina reads by using an approach similar to that described in the previous section. Results showed ~400 putative gene arrays in this strain, of which 285 (71%) have only two copies of the duplicated gene (Fig. 5; Table 2; Supplementary Data 4). Gene arrays with a relatively low copy number are not rare among eukaryotes^45^. This situation typically occurs in cases where the protein products are normally required in relatively large doses, such as ribosomal proteins or histones. Genes encoding such proteins has been found duplicated in other *Leishmania* species and also here in *L. panamensis*. One example of functionally related genes found to be tandemly duplicated in *L. panamensis* are those encoding most of the glycolytic enzymes. Their duplication might be associated with the finding that, in *Leishmania*, these enzymes are not only part of glycolysis but also appear to participate in a number of “moonlighting” extracellular functions, including cell adherence, hemoglobin binding and modulation of the host cell immune system^46^.

Conversely, larger tandem gene arrays are less common and are thought to be maintained during evolution in order to fulfill particular cellular demands, commonly in the presence of stressors^47^. Due to the lack of transcriptional control in *Leishmania*, increase in copy number within gene arrays has also been suggested to serve as a way of increasing the level of critical proteins^30^, such as those important for parasite survival and infectivity in the sandfly vector and the mammalian host. Several genes that are widely known to occur as relatively large tandem arrays in *Leishmania* species also appear to be multicopy in *L. panamensis* (Fig. 5). Furthermore, gene arrays seem to be similar in their haploid number of copies for both *L. braziliensis* and *L. panamensis*, including the array of genes encoding the GP63 metalloprotease, previously reported to have an increased number of copies in *L. braziliensis* when compared to other *Leishmania* species^8^,^30^. Another notable example is the gene coding for the NAD(P)H-dependent fumarate reductase (*FRD*), found to be multicopy in *L. braziliensis* and *L. panamensis* but not in all other *Leishmania* species. The distinctive increase in copy number for this gene is one of the factors that have been associated with an enhanced metastatic ability in these parasites^2^. Interestingly, we found a higher number of copies for ubiquitin and histone H3 in *L. panamensis*, although these genes are also typically multicopy in *Leishmania*.

As in other *Leishmania* species, our results show that amastins comprise the largest family of multicopy genes in *L. panamensis*, with 19 genes assembled in 10 loci (Fig. 5), but an haploid number of copies estimated to be around 80. Although the function of these surface glycoproteins is not known, they are thought to participate in the interaction with macrophages of the mammalian host due to their preferential expression in amastigotes^48^. Jackson^49^ classified the amastin genes in four subfamilies (α, β, γ and δ) and a proto-δ group. Analysis of the δ subfamily has shown that it is not only expanded in *Leishmania*, but also that some member genes vary greatly among species^49^,^50^. Accordingly, the genes we found in *L. panamensis* are more similar to those from *L. braziliensis*, with the majority of δ amastin genes clustered into subgenus-specific clades in a maximum likelihood phylogeny (Supplementary Fig. 6). In fact, for all amastin subfamilies, the genes from the two *L.* (*Viannia*) species tend to cluster together and are separated from those corresponding to *L.* (*Leishmania*) species, regardless of their shared genomic position. This strongly suggests diversifying evolution of these genes between subgenera, as opposed to concerted evolution within subgenera. The evolution of these genes is probably shaped by the different environmental and immunological niches to which parasites are exposed.

## Discussion

Completely sequencing the genome of *L.* *panamensis* allowed us to explore the genomic background of the *L.* (*Viannia*) subgenus, with previous studies traditionally relying only on the *L. braziliensis* genome. We confirmed several general characteristics described for *Leishmania* genomes and, at the same time, several subgenus-specific features. Our results are in agreement with recent paradigms regarding the extensive genome plasticity in *Leishmania*. This genome plasticity is evidenced at several levels, including mosaic aneuploidy^39^ and stochastic amplification of genomic regions mediated by homologous recombination^35^. Repetitive sequences spread throughout the genome are thought to provide favorable conditions for several types of recombinational events leading to extrachromosomal amplifications. This dynamic environment provides the parasite population with an immediate availability of genetic variants to cope with potentially deleterious conditions, such as toxic drugs or attack by host immune system factors. In addition, the lack of transcriptional control is thought to be balanced with the ability to change gene dosage by increasing the number of critical genes in tandem arrays^30^, which can be viewed as a form of “intrachromosomal amplification”. Maintaining relatively large gene arrays, however, suggests the existence of a mechanism of concerted evolution, probably with an adaptive advantage.

In addition, comparative analysis also suggests that diversification after duplication is an important source of variation in this species, in this case assuming neutral evolution. Pseudogene formation after duplication has been recognized as a relevant event responsible for species-specific differences in gene content among *Leishmania*^8^. As it was previously reported for *L. braziliensis*, we found a relatively large number of pseudogenes in *L. panamensis* as compared to *L.* (*Leishmania*) species. Although this may be explained by faster divergence and deterioration, it also suggests that specific genetic features of the *Viannia* subgenus, such as potentially active mobile elements, may contribute to a faster generation of pseudogenes. Additionally, pseudogenes generated after duplication may participate in the endogenous production of small interference RNAs (siRNAs) used in RNAi, a role that has been previously described in several eukaryotes, including *T. brucei*^51^.

We found relatively large sequence divergence when comparing several orthologs from *L.* (*Viannia*) species to those from *L.* (*Leishmania*), a situation that has often resulted in the *L.* (*Viannia*) orthologs clustered into different groups or considered to be different genes. This seems to be particularly relevant for proteins involved in surface components or in their modification, which vary significantly among species. Acting as an interface between the parasite and its hosts, surface components are largely responsible for differences in life cycle, range of vectors, tissue tropism and infective capabilities among species of *Leishmania*^26^,^52^,^53^,^54^. Differences in genes involved in LPG side chain modifications may cause a different pattern of glycosylation of this surface glycoconjugate, which in turn may alter the way in which *Viannia* parasites develop in the digestive tract of the insect vector. This may explain the additional step of development in the insect hindgut, a feature that was used as a criterion to define the *Viannia* subgenus. Furthermore, these differences in surface glycolipids, together with divergence and/or copy number variation in several critical genes — including those encoding GP63, 3-mercaptopyruvate sulfurtransferase, glutathione reductase and NAD(P)H-dependent fumarate reductase — may enhance parasite survival and metastatic abilities, promoting the development of more severe disease outcomes, such as the mucocutaneous presentation.

On the other hand, the finding of TATE-related sequences in internal positions of chromosomes, as well as repetitive sequences comprising predicted protein-coding genes with reverse transcriptase domains, suggest that the impact of mobile elements — either autonomous, non-autonomus or extinct — may be stronger than previously suspected in *L.* (*Viannia*). The presence of these elements, together with the RNAi activity and several other features considered to be specific of *L.* (*Viannia*) species, resemble traits that have been typically associated with *Trypanosoma* species. This supports the previously formulated hypothesis of an early divergence of the *Viannia* subgenus during the evolution of the *Leishmania* genus^55^,^56^, although alternative scenarios have been suggested^57^.

The sequence of the *L. panamensis* genome is a valuable resource to understand the biology and evolution of the *L.* (*Viannia*) subgenus. In addition, it provides the foundation for future studies regarding epidemiology, pathogenesis and drug resistance in countries where this parasite is a notable causative agent of leishmaniasis, including Panama and Colombia.

## Methods

### Genome sequencing

High-quality genomic DNA was extracted from the PSC-1 strain (MHOM/PA/1994/PSC-1) of *L. panamensis*, originally isolated from a skin lesion on the arm of a male subject in Panama. Genomic DNA was extracted from stationary phase promastigotes using a commercial salting out procedure as recommended by the manufacturer (Wizard Genomic DNA purification kit, Promega). Size check, integrity and presence of contaminants in the DNA samples were assessed through gel electrophoresis. DNA concentration was estimated by the picogreen method using Victor 3 fluorometry (PerkinElmer). DNA purity was measured using a NanoDrop 2000 spectrophotometer (Thermo Scientific). Two libraries were prepared for 454 pyrosequencing from 25 μg of genomic DNA, one with shotgun (single-end) reads and the other with mate-paired reads with a median insert size of 8 kb, respectively following the GS rapid library and the GS 8-kb span paired end library preparation protocols from Roche. Genomic DNA was fragmented by nebulization in the case of the shotgun read library, or in a HydroShear apparatus (Genomic Solutions Inc.), followed by circularization and nebulization in the case of the mate-paired library. Fragment size was experimentally confirmed by using a DNA 12000 LabChip with a 2100 Bioanalyzer (Agilent Technologies). Both libraries were then sequenced on a GS-FLX Titanium instrument. An additional library was prepared from 2 μg of genomic DNA by using the TruSeq DNA HT sample preparation protocol (Illumina), and then sequenced in a HiSeq 2000 instrument for a total throughput of 5 Gb.

### Reference genomes

Four reference genomes were used at different stages of this work, corresponding to *L. major* strain Friedlin^58^ (version 6.1), *L. infantum* strain JPCM5^8^ (version 5.0), *L. braziliensis* strain M2904^8^ (version 3.0) and *L. mexicana* strain U1103^30^ (version 5.0). All genomes were downloaded from the FTP site of the Wellcome Trust Sanger Institute (UK) (ftp.sanger.ac.uk/pub/pathogens/Leishmania/).

### *De novo* assembly, post-assembly improvements and short read mapping

The 454 reads were assembled *de novo* by using Newbler^13^ (version 2.6), with the built-in gap-filling and heterozygotic modes enabled (-scaffold and -het flags, respectively). The use of these modes was chosen after several tests to assess their beneficial effect on the assembly metrics. Only contigs larger than 500 bp were used during scaffolding and the subsequent steps in assembly.

The Illumina reads were used to validate the *de novo* assemblies with REAPR^14^. After running REAPR, iCORN^15^ was used to detect and correct errors derived from the 454 pyrosequencing using information from the Illumina reads. The Mauve Contig Mover^59^ was used to initially order the *de novo* fragments against the *L. braziliensis* chromosomes. Each group of fragments assigned to particular *L. braziliensis* chromosomes was submitted independently to ABACAS^16^ to contiguate them into individual pseudochromosomes, based on comparison at the nucleotide level.

Sequence reads were mapped back to the assembled pseudochromosomes or reference chromosomes by using SMALT (version 0.7.2) (https://www.sanger.ac.uk/resources/software/smalt/), with the parameters suggested in the manual for each type of read.

### Annotation of protein-coding genes and non-coding features

RATT^17^ was used to transfer the annotated genes from the genomes of *L. major* and *L. braziliensis*. In addition to RATT, Artemis^60^ was used for *de novo* prediction of open reading frames (ORFs) larger than 225 bp correlating to the expected codon usage of other *Leishmania* species, taking into account the suggestions given by Aggarwal *et al*.^61^ ORFs with a codon usage correlation score lower than 55 were discarded. The three lines of evidence for gene models were manually revised and combined during a three-way comparison of the corresponding genomes using the Artemis Comparison Tool (ACT)^60^. Pseudogenes were identified based on frameshifts or in-frame stop codons disrupting the corresponding ORFs for the transferred genes, in cases where these artifacts could be confirmed in the majority of reads mapping to the corresponding loci.

Non-coding RNA genes were predicted by scanning the sequences against the Rfam database^62^ (version 11.0), using the rfam_scan.pl script included in the distribution. tRNAscan-SE^63^ (version 1.21) was also used to predict tRNA genes.

A combined strategy was used to identify repetitive regions. First, RepeatScout^64^ was used for *de novo* detection of repeat families; then, a sequence similarity search against RepBase Update^65^ (volume 18, issue 9) was performed with RepeatMasker (http://www.repeatmasker.org), both with the default options. Families predicted *de novo* were manually correlated to hits produced by RepeatMasker, and to repeat families previously described in trypanosomatids, on the basis of sequence similarity and chromosomal location.

### Ortholog clustering and functional analysis

The OrthoMCL web assignment tool was used to assign the *L. panamensis* predicted proteins to the ortholog groups pre-defined in the OrthoMCL-DB database^66^ (ortholog ID version 5). Functional analysis of the annotated gene models was performed by using Blast2GO^67^ and InterProScan^68^ (version 5). Gene Ontology (GO) terms and Enzyme Commission (EC) numbers were obtained from the predicted domains whenever possible. Additionally, EC numbers that could not be inferred from domain architecture were transferred from orthologous annotations in other *Leishmania* genomes. Blast2GO was also used to perform the GO enrichment analysis.

### Chromosome somy and gene copy number variation analysis

SAMtools^69^ depth (version 0.1.19) was used to record the read depth per base along the assembled pseudochromosomes. These values were then used to compute the median read depth for each chromosome. To obtain an estimate of chromosome somy, the values of median read depth for each chromosome were normalized dividing by the median read depth expected for a monosomic chromosome. This value in turn was calculated from the most frequent median read depth among all the chromosomes, which was arbitrarily considered to correspond to a disomic state.

Read depth for annotated features was computed as described above, but submitting an additional file to SAMtools with the corresponding annotations in BED format. The haploid copy number for each feature was estimated by the ratio between its median read depth and the median read depth of its corresponding chromosome. For genes within the same ortholog group and located in the same chromosome, the total haploid copy number was considered to be the sum of their individual estimates.

## Author Contributions

A.L., C.M.R. and R.L. performed the experiments, analyzed the data and wrote the paper; G.D.V. and F.J.A. helped in the manual revision of gene models during annotation; R.L. conceived and directed the project.

## Additional information

**Accession codes**: This project has been registered in NCBI as bioproject PRJNA235344. The raw reads have been deposited in the Sequence Read Archive (SRA) under the accession codes SRX681913, SRX681914 and SRX681983. The assembled and annotated pseudochromosomes have been deposited in GenBank under the accession codes CP009370 to CP009404.

## Supplementary Material

Supplementary Information Supplementary Information

Supplementary Information Supplementary Data 1

Supplementary Information Supplementary Data 2

Supplementary Information Supplementary Data 3

Supplementary Information Supplementary Data 4

## Figures and Tables

**Table 1:** Summary of general statistics for the five *Leishmania* genomes considered in this study

Feature	*L. major* Friedlin	*L. infantum* JPCM5	*L. mexicana* U1103	*L. braziliensis* M2904	*L. panamensis* PSC-1
Number of chromosomes	36	36	34	35	35
Chromosome size range (Mb)	0.27–2.68	0.28–2.67	0.27–3.34	0.23–2.69	0.27–2.61
Total size (Mb)	32.85	31.92	30.94	31.24	30.69
GC content (%)	59.72	59.58	59.72	57.72	57.56
N content (%)	<0.01	0.06	0.11	0.26	2.28
Number of gaps	9	440	410	876	553
Protein-coding genes	8,400	8,199	8,106	8,175	7,748*
Pseudogenes	89	61	99	195	185
Non-coding RNA genes	893	136	96	109	163
Transfer RNA	83	67	83	66	74
Ribosomal RNA	63	11	13	6	17
Small nuclear RNA	6	8	-	7	5
Small nucleolar RNA	741	50	-	30	54

*This lower number of protein-coding genes annotated in our *L. panamensis* assembly, when compared to all other *Leishmania* genomes, is due to a relatively large number of genes organized in tandem arrays, whose redundant copies could not be fully assembled by using 454 reads. In contrast, some of these arrays could be assembled in the genomes of *L. major*, *L. infantum*, *L. mexicana* and *L. braziliensis*, as those were sequenced by using traditional Sanger sequencing.

**Table 2:** Gene arrays with more than four estimated copies per chromosome whose function could be inferred

Predicted product	Estimated haploid number	Number assembled	Chromosome
Amastin surface glycoprotein^a^	80	19	8, 20, 34, 30
Histone H3	34	1	16
β-Tubulin	32	1	33
GP63 surface metalloprotease (leishmanolysin)	28	4	10
TATE-associated hypothetical protein^b^	28	4	Several
Tuzin	26	2	8, 20
Ubiquitin	22	1	31
α-Tubulin	21	1	13
NAD(P)H-dependent fumarate reductase (FRD)	16	4	34
Elongation factor 1 α (EF1A)	15	1	17
Autophagy-related protein 8 (ATG8)	13	1	9
Kinesin	13	2	20
Peptidase M20	12	1	33
Heat-shock protein 83 (HSP83)	11	1	33
Autophagy-related protein 8 (ATG8)	10	1	19
Glucose transporter (GT1)	10	1	35
Phosphoglycan β1,3-galactosyltransferase (SCG)	8	1	2
Ubiquitin-conjugating enzyme (UBC)	8	1	20
Receptor-type adenylate cyclase (RAC)	7	3	17
ABC transporter (ABCA)	6	5	11
ATP dependent DEAD-box helicase (RH)	6	1	20
Folate/biopterin transporter (FT1)	6	3	10
Heat-shock protein 70 (HSP70)	6	2	28
Peptidase M3 (Dipeptidylcarboxypeptidase)	6	1	27
Phospholipid/glycerol acyltransferase (PGAT)	6	1	29

^a^Including all the amastin loci.

^b^Collectively refers to all the hypothetical coding sequences putatively associated with TATEs, and generally located in the sub-telomeric region of several chromosomes.
