# Supplementary material for: The genome of Leishmania panamensis: insights into genomics of the L. (Viannia) subgenus
Source: Sci Rep. 2015 Feb 24;5:8550. doi: 10.1038/srep08550 (PMC4338418; doi:10.1038/srep08550)
Supplement: Supplementary Information [file srep08550-s1.pdf]

SUPPLEMENTARY INFORMATION

The genome of *Leishmania panamensis*: insights into genomics of the *L*. (*Viannia*) subgenus.

Alejandro Llanes, Carlos Mario Restrepo, Gina Del Vecchio, Franklin José Anguizola, Ricardo Lleonart

Supplementary Table 1. Summary of statistics per assembly stage.

Supplementary Figure 1. Errors corrected by iCORN during the first six iterations.

Supplementary Figure 2. Layout of fragments in the *L. panamensis* assembly.

Supplementary Figure 3. Distribution of annotated genetic features along the *L. panamensis* chromosomes.

Supplementary Figure 4. Distribution of read depth along all the *L. panamensis* PSC-1 chromosomes.

Supplementary Figure 5. Distribution of read depth and GC content in four chromosomes exhibiting variations in somy or coverage.

Supplementary Figure 6. Phylogenetic tree of amastin genes.

Supplementary Data 1. Genes in non-syntenic segments between the chromosomes of *L. panamensis* strain PSC-1 and *L. braziliensis* strain M2904 (Excel file).

Supplementary Data 2. Ortholog groups differing in at least one of the species considered in this study (Excel file).

Supplementary Data 3. Read depth statistics and estimated somy per chromosome (Excel file).

Supplementary Data 4. Putative gene arrays in the *L. panamensis* PSC-1 genome (Excel file).

Supplementary Table 1. Summary of statistics per assembly stage.

Assembly stage	Count	Total size (bp)	N50 (bp)	L50	GC (%)	N (%)	Gaps
***De novo* assembly**							
Scaffolds	108	30,893,356	673,840	16	57.56	2.29	637
Contigs (>= 500 bp)	1,978	30,153,586	46,765	186	57.49	< 0.01	2
in scaffolds	1,689	-	-	-	-	-	-
not in scaffolds	289	-	-	-	-	-	-
Final assembly[(1)]	397	31,142,890	673,840	16	57.56	2.27	639
**Validation with REAPR**							
Validated assembly	471	30,986,015	554,718	18	57.56	2.31	562
Scaffolds fragments	182	-	-	-	-	-	-
**Error correction with iCORN**							
Corrected assembly	471	30,984,966	554,684	18	57.56	2.31	562
**Contiguation with ABACAS**							
Pseudochromosomes	35	30,688,794	-	-	57.56	2.28	553

[(1)] The result of pooling together the scaffolds and the contigs not assigned to scaffolds.

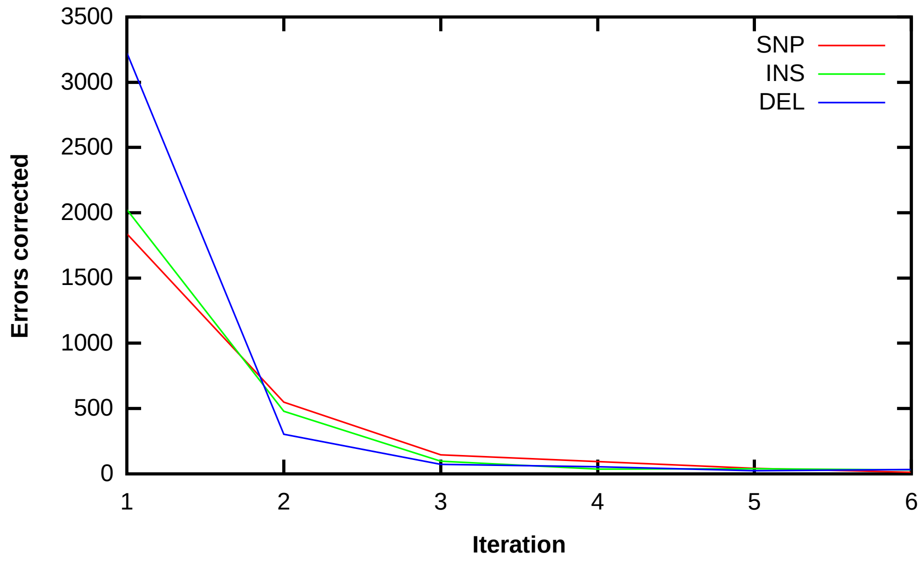

Supplementary Figure 1. Errors corrected by iCORN during the first six iterations. iCORN detects and corrects 454 pyrosequencing errors such as single-base changes (SNP) and small insertions (INS) or deletions (DEL). Errors are accurately identified by comparing the coverage of perfectly mapping reads at the corrected position between two consecutive iterations, corrections that reduce the read coverage at that position are rejected. The program was run for 10 iterations, after which the number of discrepancies corrected was similar to the number of rejected corrections.

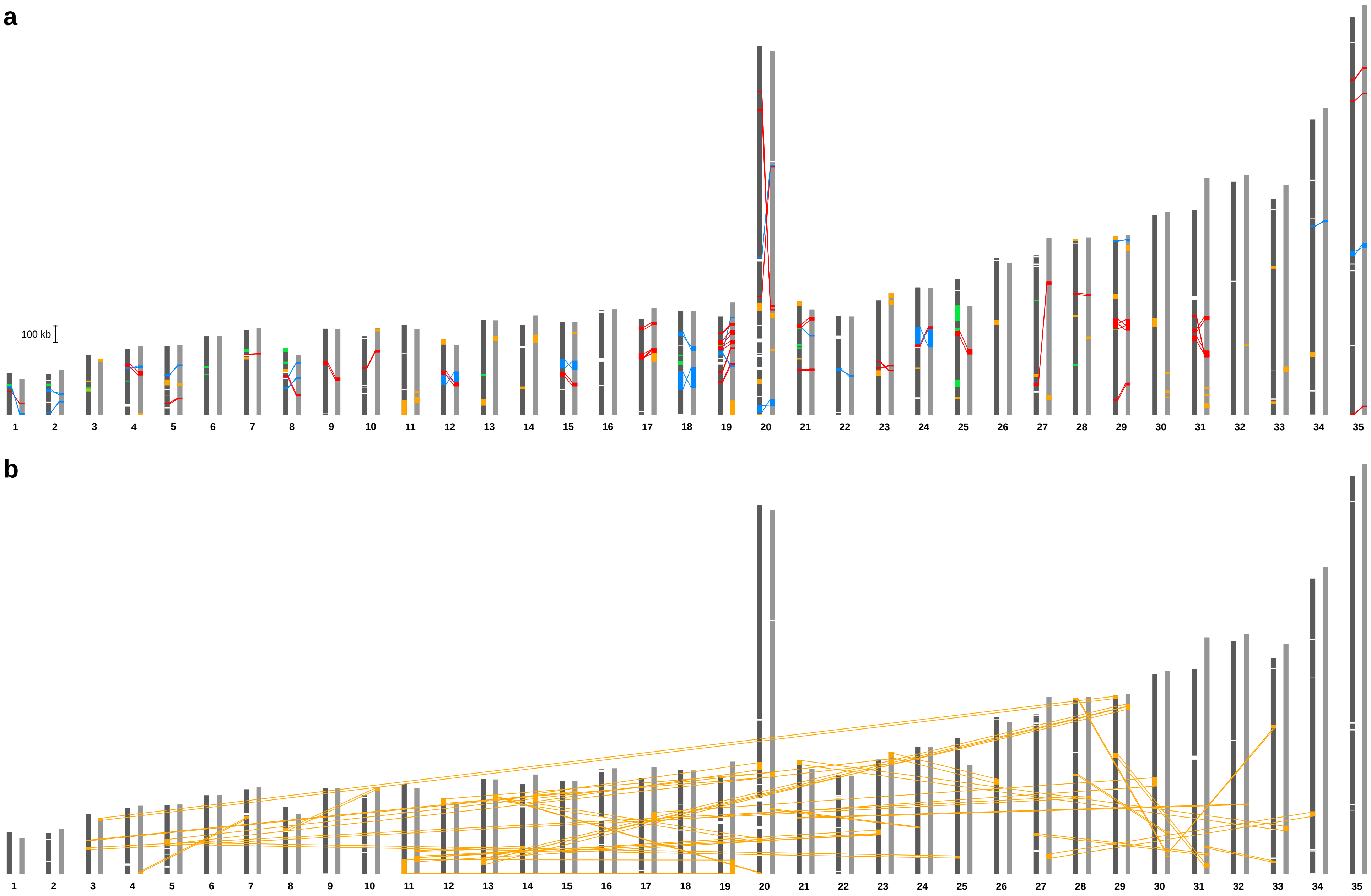

Supplementary Figure 2. Layout of fragments in the *L. panamensis* assembly. (a) *L. panamensis* pseudochromosomes (dark gray) are plotted aside the *L. braziliensis* chromosomes (light gray). Blank spaces indicate contiguation gaps. Non-syntenic segments are highlighted in red for local transpositions, blue for local inversions, orange for segments located in different chromosomes, and green for segments whose homologs in *L. braziliensis* chromosomes were removed after Rogers et al. (ref. 30), based on suspected incorrect assembly. **(b)** Similar to **(a)** but with lines connecting the segments located in different chromosomes.

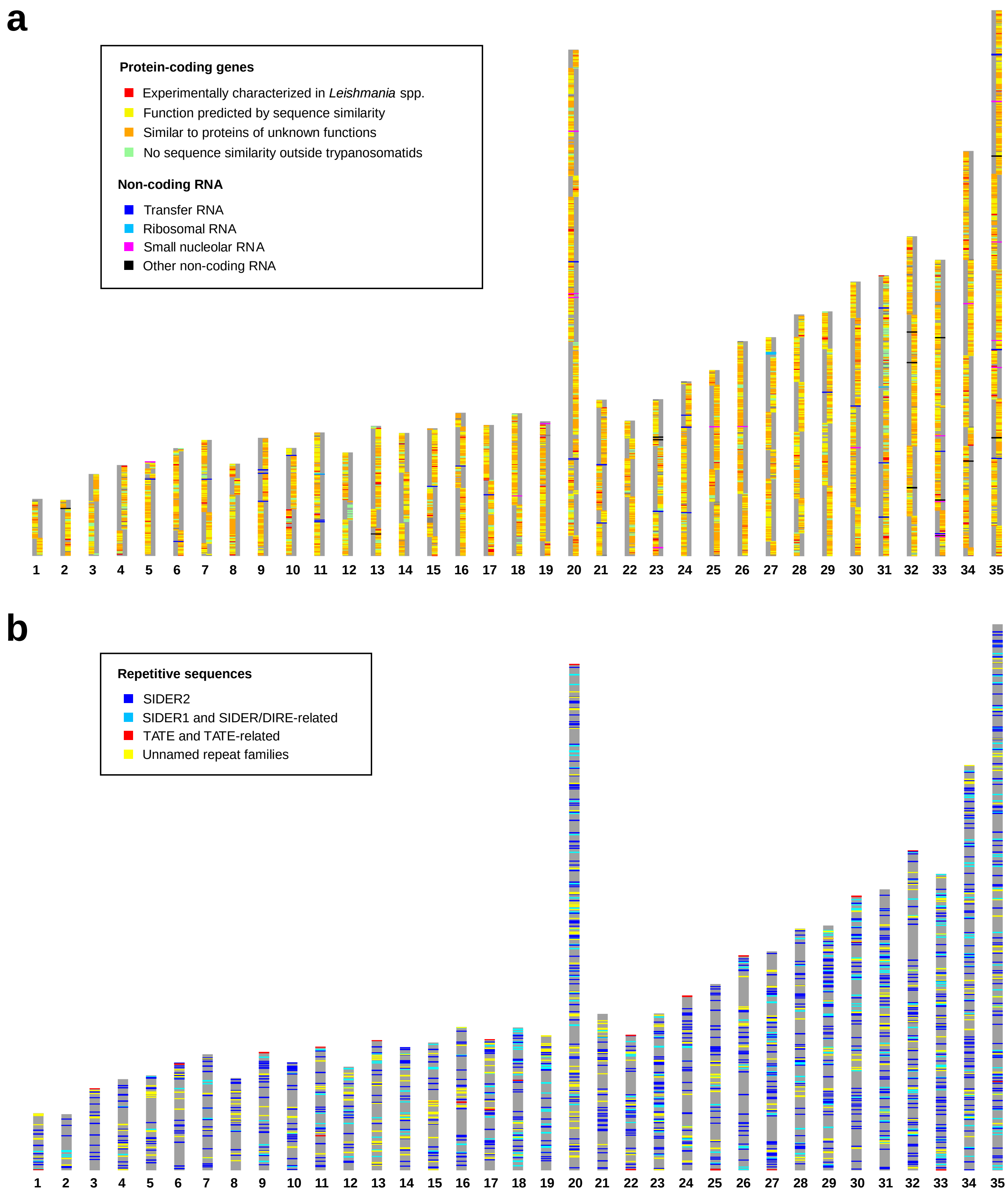

Supplementary Figure 3. Distribution of key features along the *L. panamensis* chromosomes. (a) Protein-coding and RNA genes. **(b)** Repetitive sequences.

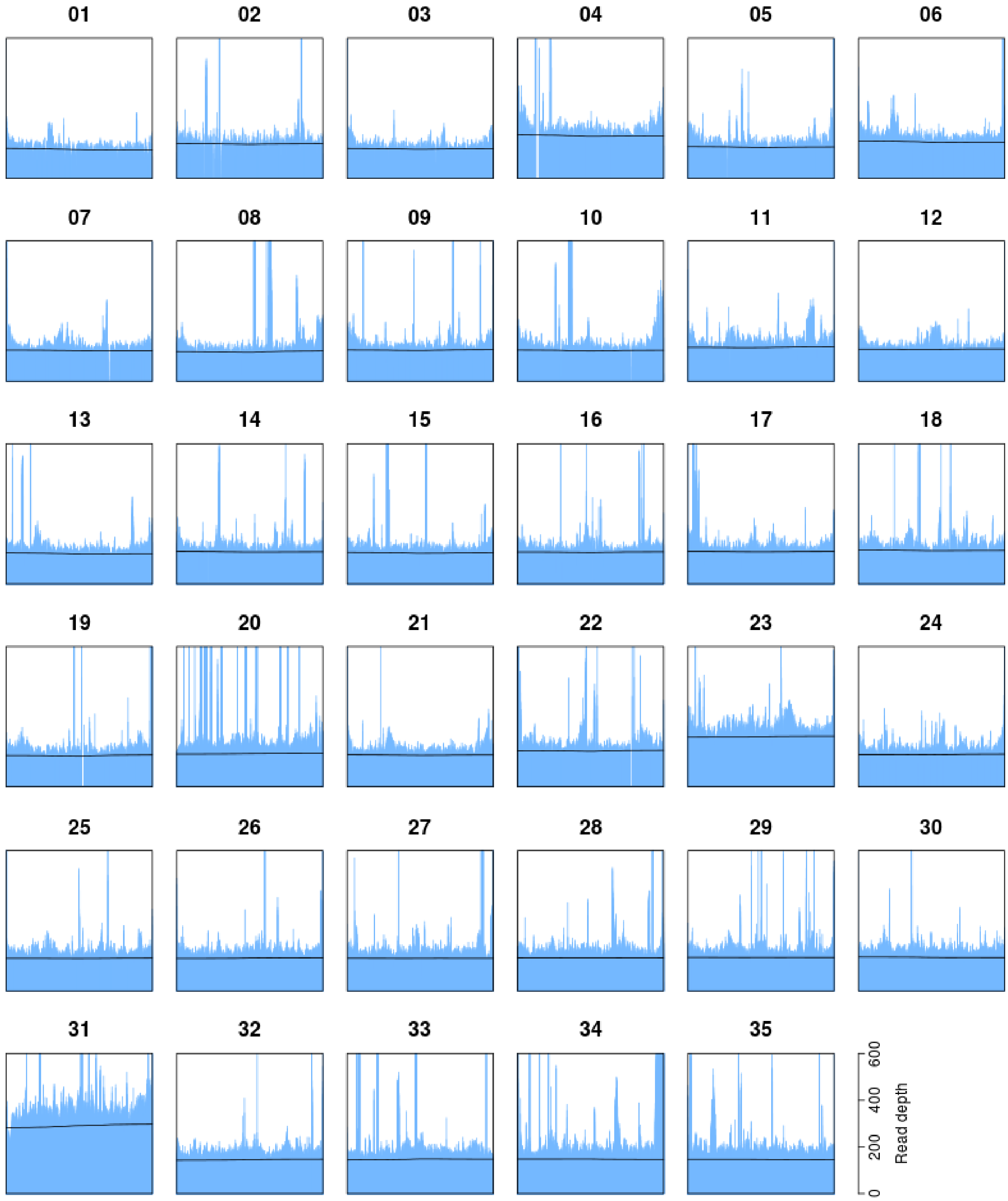

Supplementary Figure 4. Distribution of read depth along all the *L. panamensis* PSC-1 chromosomes. Raw read depth per position is plotted in light blue. The black line shows the result of applying a local polynomial regression fitting algorithm to smooth these values, using the R loess function. This line approximates to the calculated median read depth for each chromosome. All plots use a fixed length for the *x* axis, regardless of the actual chromosome length.

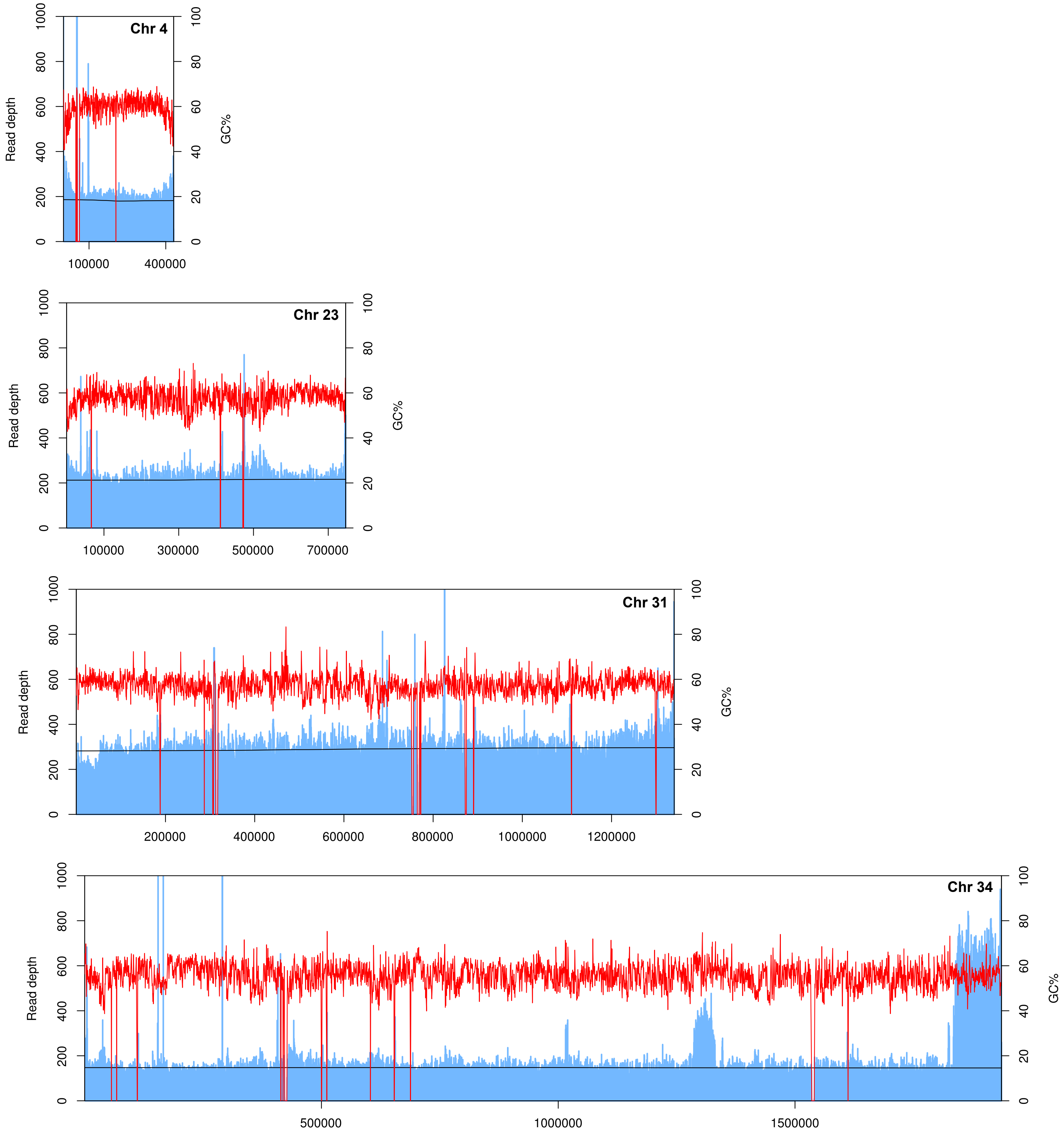

Supplementary Figure 5. Distribution of read depth and GC content in four chromosomes exhibiting variations in somy or coverage. Raw read depth (light blue) and GC% (red) are plotted along the sequence of each chromosome, both averaged over windows of 500 bp. As in Supplementary Figure 4, a local polynomial regresion fitting algorithm was used to smooth the read depth values, resulting in the black line, which approximates to the median read depth for each chromosome.

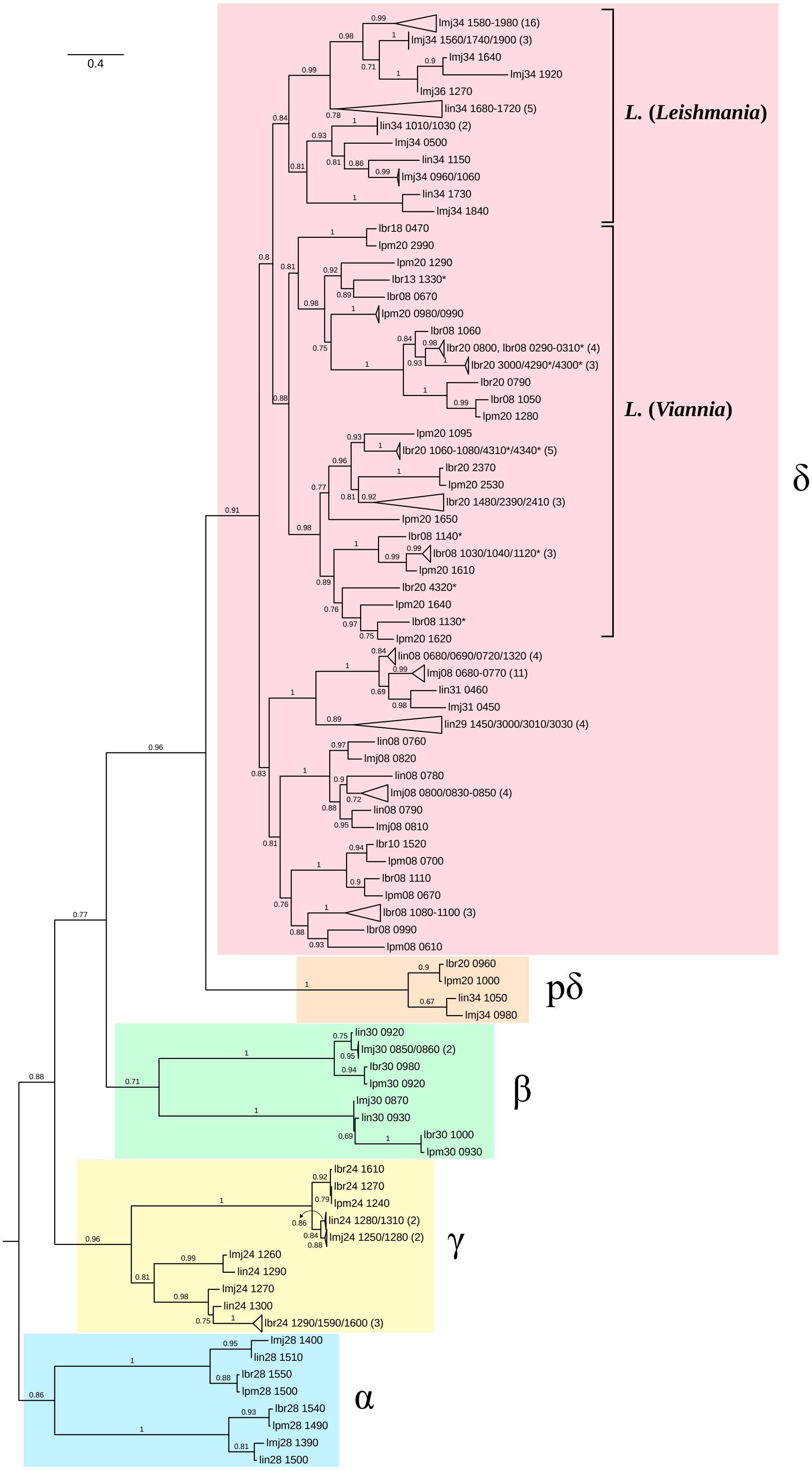
0.4
L. (Leishmania)
L. (Viannia)
δ
pδ
β
γ
α
lmj34 1580-1980 (16)
lmj34 1560/1740/1900 (3)
lmj34 1640
lmj34 1920
lmj36 1270
lin34 1680-1720 (5)
lin34 1010/1030 (2)
lmj34 0500
lin34 1150
lmj34 0960/1060
lin34 1730
lmj34 1840
lbr18 0470
lpm20 2990
lpm20 1290
lbr13 1330*
lbr08 0670
lpm20 0980/0990
lbr08 1060
lbr20 0800, lbr08 0290-0310* (4)
lbr20 3000/4290*/4300* (3)
lbr20 0790
lbr08 1050
lpm20 1280
lpm20 1095
lbr20 1060-1080/4310*/4340* (5)
lbr20 2370
lpm20 2530
lbr20 1480/2390/2410 (3)
lpm20 1650
lbr08 1140*
lbr08 1030/1040/1120* (3)
lpm20 1610
lbr20 4320*
lpm20 1640
lbr08 1130*
lpm20 1620
lin08 0680/0690/0720/1320 (4)
lmj08 0680-0770 (11)
lin31 0460
lmj31 0450
lin29 1450/3000/3010/3030 (4)
lin08 0760
lmj08 0820
lin08 0780
lmj08 0800/0830-0850 (4)
lin08 0790
lmj08 0810
lbr10 1520
lpm08 0700
lbr08 1110
lpm08 0670
lbr08 1080-1100 (3)
lbr08 0990
lpm08 0610
lbr20 0960
lpm20 1000
lin34 1050
lmj34 0980
lin30 0920
lmj30 0850/0860 (2)
lbr30 0980
lpm30 0920
lmj30 0870
lin30 0930
lbr30 1000
lpm30 0930
lbr24 1610
lbr24 1270
lpm24 1240
lin24 1280/1310 (2)
lmj24 1250/1280 (2)
lmj24 1260
lin24 1290
lmj24 1270
lin24 1300
lbr24 1290/1590/1600 (3)
lmj28 1400
lin28 1510
lbr28 1550
lpm28 1500
lbr28 1540
lpm28 1490
lmj28 1390
lin28 1500

Supplementary Figure 6. Phylogenetic tree of amastin genes. ClustalW was used to align the protein sequences of 148 representative amastin genes from *L. major* (lmj), *L. infatum* (lin), *L. braziliensis* (lbr) and *L. panamensis* (lpm). This maximum likelihood (ML) phylogeny was constructed with PhyML 3.0 using the Whelan and Goldman (WAG) model and the approximate likelihood ratio test (aLRT) for branch support. Groups involving genes from the same species and located in the same chromosomes were compressed for simplicity. The gene IDs and the number of genes are indicated near each compressed group. Asterisks indicate previously subtelomeric *L. braziliensis* genes that were further removed from the assembled chromosomes, therefore the chromosome numbers in their IDs are not reliable.
